# Population Genetic Structure and Isolation by Distance of *Helicobacter pylori* in Senegal and Madagascar

**DOI:** 10.1371/journal.pone.0087355

**Published:** 2014-01-30

**Authors:** Bodo Linz, Clairette Romaine Raharisolo Vololonantenainab, Abdoulaye Seck, Jean-François Carod, Daouda Dia, Benoit Garin, Rado Manitrala Ramanampamonjy, Jean-Michel Thiberge, Josette Raymond, Sebastien Breurec

**Affiliations:** 1 Department of Biochemistry and Molecular Biology, Pennsylvania State University, University Park, Pennsylvania, United States of America; 2 Laboratoire d’Anatomie et de Cytologie Pathologiques, Institut Pasteur, Antananarivo, Madagascar; 3 Laboratoire de Biologie Médicale, Institut Pasteur, Dakar, Senegal; 4 Centre de Biologie Clinique, Institut Pasteur, Antananarivo, Madagascar; 5 Centre Hospitalier Le Dantec, Département de Gastro-entérologie, Dakar, Senegal; 6 Laboratoire de Bactériologie expérimentale, Institut Pasteur, Antananarivo, Madagascar; 7 Centre Hospitalier Universitaire Joseph Ravoahangy Befelatanana, Département de Gastro-entérologie, Antananarivo, Madagascar; 8 Plate-forme Génotypage des Pathogènes et Santé Publique, Institut Pasteur, Paris, France; 9 Unité Postulante Pathogenèse de Helicobacter, Institut Pasteur, Paris, France; 10 Institut Pasteur, Laboratoire de Biologie médicale, Bangui, République Centrafricaine; University of Aberdeen, United Kingdom

## Abstract

*Helicobacter pylori* has probably infected the human stomach since our origins and subsequently diversified in parallel with their human hosts. The genetic population history of *H. pylori* can therefore be used as a marker for human migration. We analysed seven housekeeping gene sequences of *H. pylori* strains isolated from 78 Senegalese and 24 Malagasy patients and compared them with the sequences of strains from other geographical locations. *H. pylori* from Senegal and Madagascar can be placed in the previously described HpAfrica1 genetic population, subpopulations hspWAfrica and hspSAfrica, respectively. These 2 subpopulations correspond to the distribution of Niger-Congo speakers in West and most of subequatorial Africa (due to Bantu migrations), respectively. *H. pylori* appears as a single population in Senegal, indicating a long common history between ethnicities as well as frequent local admixtures. The lack of differentiation between these isolates and an increasing genetic differentiation with geographical distance between sampling locations in Africa was evidence for genetic isolation by distance. The Austronesian expansion that started from Taiwan 5000 years ago dispersed one of the 10 subgroups of the Austronesian language family via insular Southeast Asia into the Pacific and Madagascar, and hspMaori is a marker for the entire Austronesian expansion. Strain competition and replacement of hspMaori by hpAfrica1 strains from Bantu migrants are the probable reasons for the presence of hspSAfrica strains in Malagasy of Southeast Asian descent. hpAfrica1 strains appear to be generalist strains that have the necessary genetic diversity to efficiently colonise a wide host spectrum.

## Introduction

The association between *Helicobacter pylori* and man is very old: humans have probably been infected with *H. pylori* since their origins [1]. Like human mitochondrial DNA (mtDNA), the global phylogeny of *H. pylori* sequences consists of two super-lineages. The first contains mtDNA haplogroup L0, which is found predominantly in hunter-gatherers in southern Africa, the San, who are the natural hosts of the *H. pylori* super-lineage hpAfrica2. The other super-lineage contains the human mtDNA haplogroups L1–L6, which correspond to other *H. pylori* populations [1]. *H. pylori* accompanied modern humans during their migration out of Africa about 60,000 years ago, and mirrors the human pattern of decreased genetic diversity with distance from Africa [2]. Geographical separation plus founder effects have resulted in distinct bacterial populations with specific geographical distributions [1]–[9]: hpEurope (present in Europe, the Middle East and west and South Asia), hpNEAfrica (northeast Africa), hpAfrica1 (western, central and southern Africa), hpAfrica2 (southern Africa), hpAsia2 (northern India, Bangladesh, Thailand and Malaysia), hpSahul (in Australian Aboriginals and Papua New Guineans) and hpEastAsia with the subpopulations hspEAsia (in East Asians), hspMaori (in Taiwanese aboriginals, Melanesians and Polynesians) and hspAmerind (in Native Americans).

The specific geographic distribution and ethnic association of the *H. pylori* genetic populations reflect numerous ancient and historic human migrations [10] which established *H. pylori* sequences as a useful genetic marker to solve controversial issues in human population history. For example, *H. pylori* sequences revealed an ancient migration from India to continental Southeast Asia [9] that had not been discerned from human genetic markers. Work on *H. pylori* from Pacific islanders contributed to resolving the dispute about the origins and trajectory of Austronesian expansion [8]. According to archaeologists, agriculturists spread from Taiwan via insular and coastal Melanesia into the Pacific, as marked by the Lapita cultural complex, including red-slipped pottery, Neolithic tools, chickens, pigs and farming [11]. This trajectory was supported by linguists, because the topology of the language tree of the Austronesian language family is compatible with the conclusion that Taiwan is at the origin of the expansion [12]. Although some human geneticists favour insular Southeast Asia as the source [13], genetic patterns in DNA sequences of the *H. pylori* subpopulation hspMaori provide strong genetic support for a Taiwanese origin of Austronesian speakers [8]. *H. pylori* sequences further showed patterns of genetic variability that distinguished Buddhists and Muslims, the two major ethnic communities in Ladakh in northern India, whereas traditional human genetic markers, such as microsatellites and the hyper-variable region of mtDNA, were unable to differentiate these populations [4].

Data on the genetic structure of *H. pylori* in Africa are scarce compared to other world regions [1]–[3], [14]. Yet, this information is important for reconstructing human evolutionary history [2], [4], [8], [9] and for interpreting geographical differences in the incidence of gastric cancer, as genetic background might be a marker for virulence factors directly involved in clinical outcome [9]. We analysed the genetic diversity of 78 *H. pylori* strains isolated from five ethnic groups in Senegal (West Africa) and 24 strains from Madagascar, an island in the western Indian Ocean about 600 km off the African coast. We attempted to differentiate the human ethnicities on the basis of their *H. pylori* and to gain additional insight into human population history in Africa by studying the genetic diversity of *H. pylori*.

## Materials and Methods

### Strains and Ethics Statement

Gastroduodenal endoscopy was performed at the gastroenterology departments of Le Dantec Hospital in Dakar (Senegal) and of Befelatanana Hospital in Antananarivo (Madagascar) in 2007 and 2008, with the permission of the National Ethics Committee of Senegal (“Comité National d’Éthique pour la Recherche en Santé”) (ethics certificate 02612/MSPM/DS/DER) and of the National Ethics Committee of the Ministry of Healthand Family Planning of Madagascar (ethics certificate 006/SANPF/2007). Informed written consent was received from all participants.

The physician prospectively collected demographic data. All the patients were of indigenous origin, and none had received proton pump inhibitors or antibiotics during the 4 weeks before endoscopy. Three biopsy samples were taken from the antrum and three from the fundus during upper gastrointestinal tract endoscopy. One biopsy from each site was cultured for *H. pylori* isolation, and the others were fixed and processed for histological analysis.

The strains were supplemented by sequences obtained from the *H. pylori* multi-locus sequence typing web site (http://pubmlst.org/helicobacter/), as published by Falush *et al.* 2003 [3], Wirth *et al.* 2004 [4], Momynaliev *et al.* 2005 [15], Linz *et al.* 2007 [2], Devi *et al.* 2007 [6], Tay *et al.* 2009 [7], Liao *et al.* 2009 [16], Moodley *et al.* 2009 [10], Breurec *et al.* 2011 [9] and Moodley et al 2012 [1]. The new sequences obtained in this study were deposited in the web site under the identification numbers 1552–1656.

### Histology

Gastric biopsy samples were studied after the usual staining, and the lesions were classified according to the updated Sydney criteria [17] and the Vienna classification for dysplasia [18].

### *H. pylori* Isolates and Genomic DNA

*H. pylori* was cultured and identified as previously described [19]. A single *H. pylori* colony from the antrum or fundus was chosen and subcultured from a primary growth. Genomic DNA was extracted with the QIAmp™ kit (Qiagen, Courtaboeuf, France).

### Data Analysis

PCR amplification and sequencing of *atpA*, *efp*, *mutY*, *ppa, trpC*, *ureI* and *yphC* were performed as previously described [2]. Strain population assignment was performed as described by Falush *et al* [3] using the “no admixture model” of Structure [20]. The linkage model in Structure was used to estimate the proportion of nucleotides derived from each ancestral population, as described elsewhere [2], [3]. A Clonal Frame analysis was performed for the inference of bacterial microevolution with 100,000 iterations followed by a burn-in period of 50,000 iterations using the scaled mutational rate *θ* set equal to Watterson’s moment estimator [21], [22]. The genealogy was sampled every 100 iterations after the initial burn-in phase. This analysis was repeated 100 times, and an 80% consensus tree of all the sampled genealogies was computed.

Pairwise *F*_ST_ values and analyses of molecular variance (AMOVA) were calculated in Arlequin [23] as described previously [24], using the Kimura 2-parameter model previously applied to *H. pylori* sequences [2]–[4], [24]. The significance of the pairwise *F_ST_* values was estimated by running 10,000 permutations, assuming no difference between the populations. Neighbour-joining trees from the pairwise *F_ST_* values were generated in Mega v4 [25]. Nucleotide diversity (π) was calculated by DnaSP as previously described [8].

In univariate analysis, the chi-square test was used to compare categorical variables. P-values <0.05 were considered to denote significant associations.

## Results

### *H. pylori* Strains from Senegal and Madagascar

The 158 patients in Senegal included in this study were from the Wolof (32.9%), Fulani (15.2%), Mande (12.7%), Serer (10.1%) and Tuculor (5.1%) ethnic groups, reflecting the ethnic diversity in Senegal. The ethnic origin was based on self-proclaimed membership of the ethnic group for at least two generations. Thirty-seven (24.1%) biopsies were obtained from patients of uncertain ethnicity or who identified themselves as offspring of mixed marriages. The infection rate with *H. pylori* (n = 126, 79.7%), based on histological analysis and bacterial culture, was comparable in all ethnicities, ranging from 75.0% in Fulani and Tuculor to 93.8% in Serer (difference not significant). The median age was 41.7 years (mean, 45.0 years; range, 18–93 years), and 55% of the patients (n = 87) were male. On the basis of the endoscopic findings, 23 patients had gastritis only, 48 had ulcerated lesions, and 11 had suspicion of neoplasia. All cases of suspected neoplasia were histologically confirmed as gastric cancer.

The infection rate among patients from Madagascar was 74.0% (54 out of 73) but only 28 biopsies were positive for *H. pylori* because of inadequate laboratory facilities. Patients were classified ethnically from their appearance: 60 (82.2%) were recognizable as tropical Southeast Asians and 13 (17.8%) as Africans. There was no significant difference in infection rate between ethnic groups. The patients’ median age was 38 years (range, 3–87 years; mean, 42.0 years), and 59% (n = 43) were male. Endoscopic examination revealed ulcerated lesions in 10 patients (13.7%), while the other 63 (86.3%) participants had gastritis only.

Sequencing of the seven housekeeping gene fragments that were used in previous analyses [2], [8], [9] showed unambiguous sequences in 78 strains in Senegal and 24 in Madagascar. The concatenated sequences (3406 base pairs) yielded 102 unique haplotypes that contained 618 polymorphic sites. These haplotypes were compared with 160 haplotypes from other African countries and about 1000 haplotypes from other continents. Bayesian clustering algorithms implemented in the “no-admixture model” of Structure [3] assigned all 102 strains to the population hpAfrica1, all 78 strains from Senegal to the subpopulation hspWAfrica and the 24 strains from Madagascar to hspSAfrica. HpAfrica1 strains have previously been shown to be composed of 80–90% of the ancestral Africa1 population and 10–20% of the ancestral Europe2 population [2]. We used the linkage model of Structure to estimate the proportion of nucleotides derived from each of the previously identified ancestral populations [2], [3], [8] and found that the strains from Senegal and Madagascar were almost pure descendants of ancestral Africa1.

A phylogenetic analysis with Clonal Frame [22] on the haplotypes of *H. pylori* strains from Senegal and Madagascar confirmed the clustering of the haplotypes into 2 subpopulations, hspWAfrica (Senegal) and hspSAfrica (Madagascar). However, no substructure was found in either of the subpopulations that correlated with the ethnic origin or the gastroduodenal pathology (data not shown).

### Lack of Differentiation between *H. pylori* Strains from Different Ethnic Groups in Senegal

We attempted to differentiate five ethnic groups in Senegal from the genetic diversity of their *H. pylori* strains, as had been done in previous analyses of the genetic diversity of *H. pylori*in Ladakh in northern India [4] and in several ethnicities in Iran [24]. Of the 78 sequenced strains from Senegal, 25 were isolated from Wolof (32.1%), 15 from Fulani (19.2%), 14 from Mande (17.9%), 10 from Serer (12.8%) and 4 from Tuculor (5.1%). The remaining 10 strains (12.8%) were obtained from descendants of mixed marriages or people of unknown ethnic background (Table S1). We compared these strains to the 24 strains from Madagascar with East Asian (87.5%) or African (12.5%) ancestry and to other, previously described strains of the hpAfrica1 population isolated in Burkina Faso (12 strains), Algeria (3 strains), Morocco (5 strains), and the Bantu ethnicities of the Xhosa (17 strains) and the Northern Sotho in South Africa (23 strains) [3], [26]. These strains were supplemented with 25 *H. pylori* isolates from San, hunter-gatherers in southern Africa (Table S1) [1].

The *H. pylori* genetic diversity (π) within individual ethnic groups in all locations was similar (Table 1). The exceptions were hpAfrica1 bacteria from Madagascar, which showed considerably lower genetic diversity (π = 0.0232 vs 0.0269–0.0309), probably because of “bottlenecks” associated with the migration of Bantu from the African east coast carrying *H. pylori* across the Mozambique Channel to Madagascar. In contrast, the genetic diversity of the three strains from Algeria was higher (π = 0.0405), possibly because of the few isolates.

**Table 1:** Within group genetic distance and between group pair-wise F_ST_ of hpAfrica1 *H. pylori* strains from Africa.

Country/ethnicity		Haplo- types (n)	Within-group genetic distance	Pair-wise *F*_ST_ between *H. pylori* populations										
				Wolof	Serer	Fulani	Tuculor	Mande	Burkina Faso	Morocco	Algeria	Northern Sotho	Xhosa	Mada-gascar
Senegal	Wolof	25	0.0269											
	Serer	10	0.0303	0.0121										
	Fulani	15	0.0284	0.0022	0.0001									
	Tuculor	4	0.0307	0.0112	0.0009	0.0034								
	Mande	14	0.0276	0.0014	0.0055	0.0030	0.0210							
Burkina Faso		12	0.0277	**0.0499**	**0.0449**	**0.0356**	**0.0582**	**0.0660**						
Morocco		5	0.0313	**0.0912**	**0.0967**	**0.0521**	**0.0711**	**0.0999**	**0.0845**					
Algeria		3	0.0405	**0.1086**	**0.0726**	**0.0924**	**0.0545**	**0.1043**	**0.1063**	**0.0344**				
South Africa	Northern Sotho	23	0.0309	**0.1555**	**0.1457**	**0.1392**	**0.1142**	**0.1571**	**0.1417**	**0.1325**	**0.1326**			
	Xhosa	17	0.0280	**0.1739**	**0.1698**	**0.1539**	**0.1292**	**0.1814**	**0.1690**	**0.1796**	**0.1621**	**0.0479**		
Madagascar		24	0.0226	**0.2650**	**0.2698**	**0.2576**	**0.2739**	**0.2750**	**0.2546**	**0.2946**	**0.2661**	**0.1428**	**0.1104**	
San		25	0.0275	**0.2178**	**0.2100**	**0.1995**	**0.1915**	**0.2270**	**0.2025**	**0.2239**	**0.2189**	**0.1240**	**0.0939**	**0.1067**

Significant F_STs_ (*P*>0.05) are shown in bold.

**Figure 1:**
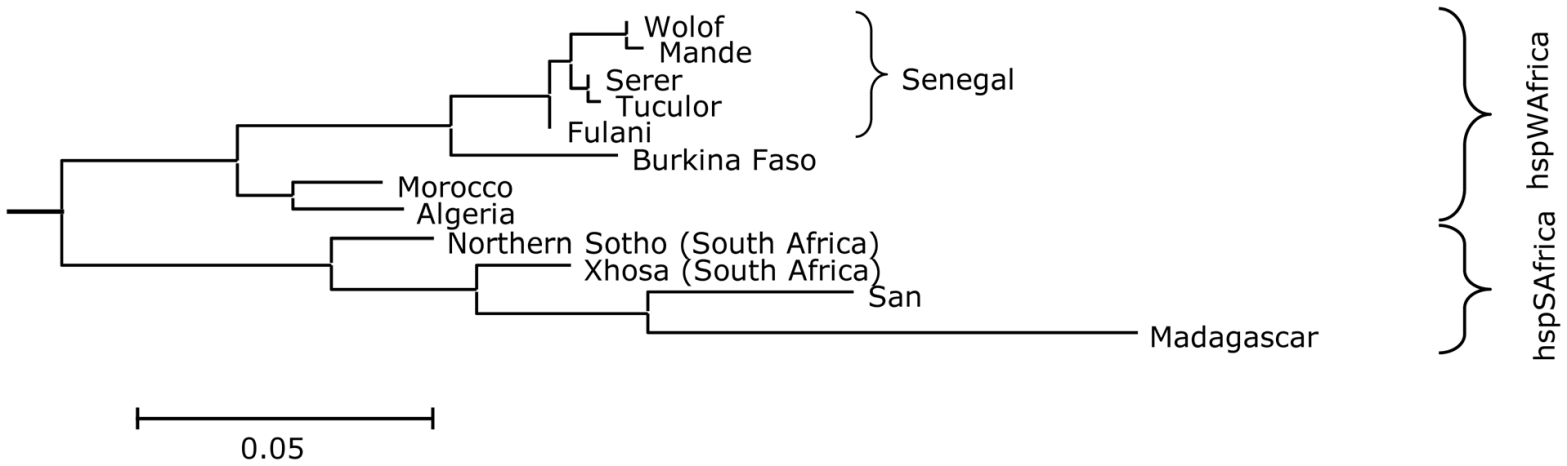
Neighbour-joining tree based on pairwise *F*_ST_ values of hpAfrica1 haplotypes from sampling locations in Africa.

We next analyzed pairwise *F*_ST_, a measure of genetic differentiation between populations, where an *F*_ST_ of zero indicates no divergence, implying free recombination between two populatins, and an *F*_ST_ of one indicates complete isolation of populations. The *F*_ST_ values from the ethnic groups in Senegal were very low and not significantly differentiated (Table 1). For example, the pairwise *F*_ST_ of 0.0121 in *H. pylori* from Wolof and Serer means that only 1.21% of the genetic variance is due to inter-population differentiation whereas 98.8% of the variance can be attributed to the intra-population component. Similarly, the net between population diversity of *H. pylori* from Senegal was close to zero (Table S2). However, the Senegalese strains significantly differed from the Burkina Faso isolates (*F*_ST_ ≈ 0.05), despite the relatively close geographical proximity of the sampling locations, their close relation in a Neighbor-joining tree (Figure1) and their low net between population diversity. The isolates from Senegal also significantly differed from all other analysed hpAfrica1 *H. pylori* populations, including those from Morocco and Algeria. In general, *H. pylori* from different geographic origins were differentiated from each other, which was also reflected in the medium net population diversity between most populations. In contrast, *H. pylori* from the same locations such as the *H. pylori* isolates from Malagasy of Southeast Asian and of African descent were not differentiated (not shown).

Structure runs of the Senegalese isolates consistently revealed the highest probability for only one population. In runs assuming *K*>1, all isolates were assigned to a single population, and none were assigned to other populations in any of the runs for 2≤ *K* ≤5, confirming a lack of difference between *H. pylori* isolates from ethnic groups in Senegal and indicating admixture between the bacteria. In contrast, Structure runs on isolates from Senegal and from Burkina Faso resulted in two populations, one for each country, except for three isolates from each geographical location that were assigned to the other.

### Isolation by Distance

**Figure 2:**
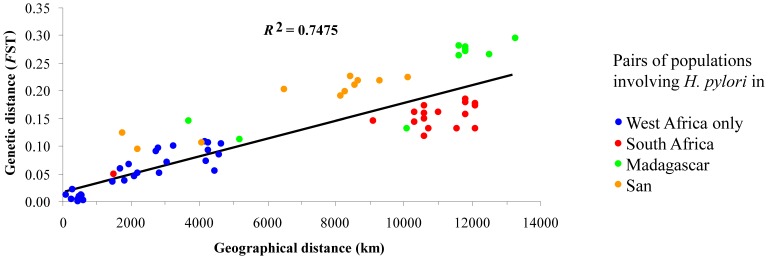
Genetic isolation by distance. The genetic distance in *H. pylori* between pairs of geographical populations (*F*_ST_) was plotted against the geographical distance between the two populations. 75% of the variance in *F*_ST_ was accounted for by geographical distance.

The lack of differentiation between isolates from the same geographic sources and the clear differentiation between isolates from different geographic locations suggested isolation-by-distance between the African isolates, similar to the previously reported isolation by distance between *H. pylori* from globally representative geographic sources [2]. However, the latter study was predominantly based on non-African *H. pylori* and involved only few isolates from sub-Saharan Africa. Therefore, we plotted the genetic distance (*F*_ST_) between hpAfrica1 *H. pylori* from sub-Saharan Africa against the geographic distance (Fig. 2). The resulting *R* ^2^ of 0.75 showed that 75% of the variance in *F*_ST_ was accounted for by geographic distance, which was considerably more than the previously determined 47% for *H. pylori* from global sources [2]. The *R* ^2^ increased to 0.79 when the isolates from the San were excluded and to 0.88 when isolates from both San and Madagascar were excluded.

## Discussion

### Isolation by Distance and Genetic Diversity

The *H. pylori* from five different ethnicities from Senegal were so similar to each other that we could not differentiate the ethnic sources based on their *H. pylori* genetic diversity. Instead, the *H. pylori* appeared as a single population at this geographic origin, indicating a long common history as well as frequent local admixture. According to archaeologists, Senegambia was populated from the north and east in several migration waves, the last being that of the Wolof, the Fulani and the Serer during the end of the first millennium AD and the early second millennium AD [27].

In contrast to the Senegalese strains, *H. pylori* from several ethnicities from Iran were sufficiently differentiated that their *F*_ST_ displayed substructure. In addition, some Iranian *H. pylori* were closely related to those from neighboring countries such as Israel, Turkey or Uzbekistan [24], reflecting frequent historical contact. However, isolates from neighbouring countries of Senegal, Mauritania to the north and Mali to the east, The Gambia in its centre, and Guinea and Guinea-Bissau to the south, are still missing to gain additional insights; the only exception is currently Burkina Faso.

Gradual genetic introgression of *H. pylori* from an outside source into one of several culturally and/or ethnically separated communities was shown to provide means to distinguish between otherwise genetically closely related groups, for example Muslims and Buddhists in Ladakh in northern India [4]. While traditional human genetic markers such as microsatellites or the hyper-variable region of the mtDNA were not able to differentiate between these communities, *H. pylori* sequences showed patterns of genetic variability that distinguished Buddhists and Muslims [4]. All the Senegalese isolates, however, belonged to the *H. pylori* population hpAfrica1. Despite the French colonization of Senegal for about a century (1854–1960), the isolates showed no traces of introgression from other *H. pylori* populations, such as hpEurope, implying only limited, if any, genetic exchange with European strains. Thus, the lack of a sufficiently different donor population for the introgression of foreign nucleotides essentially restricted the Senegalese isolates to the hpAfrica1 population, and frequent local admixture between *H. pylori* blurred potential signals that would have allowed to distinguishing the ethnicities.

Instead, the lack of differentiation between isolates from Senegal and the increasing genetic differentiation with geographical distance between sampling locations is evidence for genetic isolation by distance. 75% of the variance in *F*_ST_ was accounted for by geographical distance, considerably more than the 47% that was estimated for *H. pylori* from global sources [2]. The *R* ^2^ increased to 0.88 when the isolates from the San and from Madagascar were excluded, likely due to the elevated *F*_ST_ values in the pairwise comparisons that involved these sampling locations. The higher *F*_ST_ were likely caused by the bottlenecks that were associated with the colonization of Madagascar from the east African coast and by the transmission of hpAfrica1 from Bantu to San [1].

### Distribution of hpAfrica1 Strains in Sub-Saharan Africa by Niger-Congo Speakers

The Niger-Congo language family is distributed all over West Africa and most of subequatorial Africa [28], [29]. All Niger-Congo languages of subequatorial Africa belong to a single, low-order subgroup of the Niger-Congo language family, Bantu, which contains nearly half of the 1532 Niger-Congo languages, while most of the 176 other subfamilies are confined to West Africa. From their homeland in Nigeria and Cameroon, Bantu societies expanded east and then south over most of subequatorial Africa, distributing a variety of agricultural practices, the Bantu branch of the Niger-Congo language family and *H. pylori* of the hpAfrica1 population. This expansion, which started as early as 4000 BP, had reached its southern limit in eastern South Africa by 700 AD [29], [30]. The short time period since the beginning of the Bantu expansion only allowed the development of two closely related *H. pylori* subpopulations, hspSAfrica that resulted from the Bantu migrations and hspWAfrica that is characteristic for strains from West Africa, including Senegal [2]. Due to the slave trade, hspWAfrica *H. pylori* can be found in North and South America, particularly at high frequency among African Americans. The presence of West African *H. pylori* in Maghreb (Morocco, Algeria) probably reflects contacts between North Africa and the great sub-Saharan empires such as those of Ghana (700–1100 AD), Mali (800–1550 AD) and Songhai (1300–1600 AD), including the trade of gold, salt and slaves [29], [31].

### Strains from Madagascar

The Austronesian expansion that started from Taiwan 5000 years ago dispersed one of the 10 subgroups of the Austronesian language family along with one of several clades of the *H. pylori* population, hspMaori, via insular Southeast Asia into Melanesia and Polynesia, making hspMaori a marker for the entire Austronesian expansion [8]. Subsequent migration of a small group of Southeast Asian islanders with an effective founding population size of 30 women resulted in the settlement of Madagascar by Austronesian speakers around 840 AD [32]. They were joined around 1000 AD by Bantu migrants crossing the Mozambique Channel [33], followed by the arrival of European and Chinese settlers and Indian slaves within the past 200 years. A complex, largely unknown genetic admixture, involving mainly populations of African and Southeast Asian descent, resulted in the Malagasy we recognize today, although differences in ethnic origins between the highland and coastal regions remain evident [34], [35].

Malaysia and Indonesia, the presumed origins of the first settlers of Madagascar, were probably free of *H. pylori*, as this bacterium was introduced into the area only recently by migrants from China (hspEAsia) and India (hpAsia2 and hpEurope) [7], [9]. Therefore, the small number of Austronesian migrants probably arrived in Madagascar with few if any *H. pylori* of the population hspMaori. In contrast, the prevalence of *H. pylori* among Bantu in southern Africa is very high, affecting 80–90% of the population [36]–[38], suggesting a strong influx of hpAfrica1 *H. pylori* from the Bantu migrants. Subsequent strain competition and replacement of hspMaori by hpAfrica1 strains are the probable sources of hspSAfrica strains in Malagasy of Southeast Asian descent. Migrations from China, Europe and India within the past 200 years probably introduced other *H. pylori* populations into the area; however, we did not detect these in our study, probably because of the small number of strains. Further investigations are needed to confirm our findings based on a larger collection of strains.

### Adaptation of hpAfrica1 Strains toa wide Range of Human Hosts

Adherence of *H. pylori* to ABO/Lewis b blood group antigens in the human stomach is mediated by the blood group antigen-binding adhesion BabA [39]. Most South American natives carry the O blood group, and the long co-evolution of their indigenous *H. pylori* strains of the hspAmerind population resulted in specialist strains that show a high affinity to bind O blood group antigens but not A or B. In contrast, *H. pylori* strains from Europe and Japan appear to be generalist strains as they bind all three (A, B and O) blood group antigens. Possibly because generalist strains had a better chance of colonizing diverse niches, they appear to have outcompeted the specialist strains, as hspAmerind strains of the South American Amerindians are being replaced by European and African *H. pylori* and are absent from the Mestizos [3], [40].

Similar to Europeans, blood groups A, B and O are common among Africans, with minor differences in prevalence between individual ethnic groups [41]. Like *H. pylori* of the population hpEurope, hpAfrica1 strains seem to be generalist strains that have the necessary genetic diversity to efficiently colonise a wide host spectrum. Indeed, in addition to Bantu and other Niger-Congo speakers, hpAfrica1 *H. pylori* were found at high frequency among Europeans (40%), Cape Coloureds (85%) and San (46%) from South Africa [1], [2]. Moreover, besides the high prevalence among Malagasy of Southeast Asian origin, hpAfrica1 bacteria are spreading among South American Natives and mestizos at the cost of hspAmerind strains [40], as well as among Baka pygmies after secondary contact with their Bantu agriculturalist neighbors [14].

In conclusion, horizontal transmission among ethnically distinct groups, frequent local admixture and strain competition have probably blurred signals that might have allowed us to retrace the history of human migrations in Madagascar and Senegal. The lack of differentiation between isolates from Senegal and the increasing genetic differentiation with geographical distance between sampling locations in Africa was evidence for genetic isolation by distance.

## Supporting Information

Table S1 Sources and population assignment of the *H. pylori* strains analysed.
(DOC)

Table S2 Net between-population diversity of *H. pylori* in Senegal and other sources in Africa.
(DOC)
