# Supplementary material for: Population Genetic Structure and Isolation by Distance of Helicobacter pylori in Senegal and Madagascar
Source: PLoS One. 2014 Jan 30;9(1):e87355. doi: 10.1371/journal.pone.0087355 (PMC3907543; doi:10.1371/journal.pone.0087355)
Supplement: Table S1 — Sources and population assignment of the H. pylori strains analysed. (DOC) [file pone.0087355.s001.doc]

**Table S1. Sources and population assignment of the *H. pylori* strains analysed**

| Source | | | Number of isolates assigned to | | | | |  |
| --- | --- | --- | --- | --- | --- | --- | --- | --- |
| Country | Ethnic origin | Linguistic group | hpEurope | hpNEAFrica | hspWAfrica | hspSAfrica | hpAfrica2 | Reference |
| Iran |  | Indo-European | 125 |  |  |  |  | 24 |
| Kazakhstan | Kazakh | Indo-European | 6 |  |  |  |  | 2 |
| Turkey | Turkish | Indo-European | 18 |  |  |  |  | 2 |
| Estonia | Estonian | Indo-European | 10 |  |  |  |  | 3 |
| Finland | Finnish | Indo-European | 10 |  |  |  |  | 3 |
| Germany | German | Indo-European | 23 |  |  |  |  | 3 |
| Netherlands | Dutch | Indo-European | 5 |  |  |  |  | 2 |
| United Kingdom |  | Indo-European | 16 |  |  |  |  | 3 |
| Russia | Russian | Indo-European | 21 |  |  |  |  | 15 |
| France | French | Indo-European | 8 |  |  |  |  | 9 |
| Italy | Italian | Indo-European | 9 |  |  |  |  | 2 |
| Spain | Spanish | Indo-European | 35 |  |  |  |  | 2 |
| Spain | Basque | Indo-European | 44 |  |  |  |  | 3 |
| USA | Afro-American | Indo-European |  |  | 10 |  |  | 3 |
| India (Andhra Pradesh) | Indian | Indo-European | 23 |  |  |  |  | 6 |
| Thailand | Thai | Thai-Kadai | 6 |  |  |  |  | 2 |
| Cambodia | Khmer | Austro-Asiatic | 34 |  |  |  |  | 9 |
| Malaysia | Malay | Sino-Tibetan | 4 |  |  |  |  | 7 |
| Malaysia | Indian | Austronesian | 8 |  |  |  |  | 7 |
| Madagascar |  | Austronesian |  |  |  | 28 |  | This study |
| Philippines |  |  | 7 |  |  |  |  | 2 |
| Lebanon |  | Afro-Asiatic | 5 |  |  |  |  | 2 |
| Palestine |  | Afro-Asiatic | 11 |  |  |  |  | 2 |
| Algeria | Berber | Afro-Asiatic | 19 | 10 | 3 |  |  | Unpublished data |
| Morocco | Berber | Afro-Asiatic |  |  | 5 |  |  | 3 |
| Ethiopia | Ethiopian | Afro-Asiatic |  | 48 |  |  |  | 2 |
| Somalia | Somali | Afro-Asiatic |  | 2 |  |  |  | 2 |
| Sudan |  | Afro-Asiatic |  | 2 |  |  |  | 2 |
| Burkina Faso |  | Niger-Congo |  |  | 12 |  |  | 3 |
| Senegal | Mande | Niger-Congo |  |  | 14 |  |  | This study |
| Senegal | Fulani | Niger-Congo |  |  | 15 |  |  | This study |
| Senegal | Serer | Niger-Congo |  |  | 10 |  |  | This study |
| Senegal | Tuculor | Niger-Congo |  |  | 4 |  |  | This study |
| Senegal | Wolof | Niger-Congo |  |  | 25 |  |  | This study |
| Senegal | Unknown | Niger-Congo |  |  | 11 |  |  | This study |
| Nigeria | Kanuri | Nilo-Saharan |  | 8 |  |  |  | 2 |
| South Africa | Xhosa | Niger-Congo |  |  |  | 17 | 15 | 3 |
| South Africa | European | Indo-European | 10 |  |  | 9 | 3 | 3 |
| South Africa  South Africa  South Africa  South Africa | Cape Coloured  !Xun San  Khwe San  Khomani San | Indo-European  Khoisan  Khoisan  Khoisan |  |  | 6 | 23  13  5  7 |  | 3  1  1  1 |
