# Supplementary material for: Population Genetic Structure and Isolation by Distance of Helicobacter pylori in Senegal and Madagascar
Source: PLoS One. 2014 Jan 30;9(1):e87355. doi: 10.1371/journal.pone.0087355 (PMC3907543; doi:10.1371/journal.pone.0087355)
Supplement: Table S2 — Net between-population diversity of H. pylori in Senegal and other sources in Africa. (DOC) [file pone.0087355.s002.doc]

**Table S2. Net between-population diversity of *H. pylori* in Senegal and other sources in Africa**

|  | Country / Ethnic origin | 1 | 2 | 3 | 4 | 5 | 6 | 7 | 8 | 9 | 10 | 11 |
| --- | --- | --- | --- | --- | --- | --- | --- | --- | --- | --- | --- | --- |
| 1 | Senegal - Mande |  |  |  |  |  |  |  |  |  |  |  |
| 2 | Senegal - Fulani | 0.0001 |  |  |  |  |  |  |  |  |  |  |
| 3 | Senegal - Serer | 0.0000 | 0.0000 |  |  |  |  |  |  |  |  |  |
| 4 | Senegal - Tuculor | 0.0003 | 0.0000 | 0.0000 |  |  |  |  |  |  |  |  |
| 5 | Senegal - Wolof | 0.0000 | 0.0000 | 0.0000 | 0.0000 |  |  |  |  |  |  |  |
| 6 | Burkina Faso | 0.0020 | 0.0011 | 0.0013 | 0.0015 | 0.0015 |  |  |  |  |  |  |
| 7 | Morocco | 0.0029 | 0.0017 | 0.0031 | 0.0025 | 0.0026 | 0.0025 |  |  |  |  |  |
| 8 | Algeria | 0.0015 | 0.0019 | 0.0009 | 0.0016 | 0.0016 | 0.0019 | 0.0006 |  |  |  |  |
| 9 | South Africa - Northern Sotho | 0.0057 | 0.0051 | 0.0054 | 0.0042 | 0.0055 | 0.0051 | 0.0049 | 0.0036 |  |  |  |
| 10 | South Africa - Xhosa | 0.0063 | 0.0054 | 0.0059 | 0.0041 | 0.0060 | 0.0058 | 0.0062 | 0.0041 | 0.0015 |  |  |
| 11 | Madagascar | 0.0095 | 0.0091 | 0.0091 | 0.0085 | 0.0093 | 0.0086 | 0.0098 | 0.0066 | 0.0047 | 0.0032 |  |
| 12 | San | 0.0082 | 0.0073 | 0.0075 | 0.0064 | 0.0079 | 0.0072 | 0.0080 | 0.0062 | 0.0042 | 0.0029 | 0.0031 |
